# Species, causes, and outcomes of wildlife rehabilitation in New York State

**DOI:** 10.1371/journal.pone.0257675

**Published:** 2021-09-21

**Authors:** Melissa Hanson, Nicholas Hollingshead, Krysten Schuler, William F. Siemer, Patrick Martin, Elizabeth M. Bunting

**Affiliations:** 1 Cornell Wildlife Health Lab, New York State Veterinary Diagnostic Laboratory, College of Veterinary Medicine, Cornell University, Ithaca, New York, United States of America; 2 Center for Conservation Social Sciences, Cornell University, Ithaca, New York, United States of America; 3 New York State Department of Environmental Conservation, Albany, New York, United States of America; Universidad Miguel Hernandez de Elche, SPAIN

## Abstract

Wildlife rehabilitation is a publicly popular practice, though not without controversy. State wildlife agencies frequently debate the ecological impact of rehabilitation. By analyzing case records, we can clarify and quantify the causes for rehabilitation, species involved, and treatment outcomes. This data would aid regulatory agencies and rehabilitators in making informed decisions, as well as gaining insight into causes of species mortality. In New York State, the Department of Environmental Conservation (NYSDEC) has licensed rehabilitators since 1980 and annual reporting is required. In this study, we analyzed 58,185 individual wildlife cases that were attended by New York rehabilitators between 2012 and 2014. These encompassed 30,182 (51.9%) birds, 25,447 (43.7%) mammals, 2,421 (4.2%) reptiles, and 75 (0.1%) amphibians. We identified patterns among taxonomic representation, reasons for presentation to a rehabilitation center, and animal disposition. Major causes of presentation were trauma (n = 22,156; 38.1%) and orphaning (n = 21,679; 37.3%), with habitat loss (n = 3,937; 6.8%), infectious disease (n = 1,824; 3.1%), and poisoning or toxin exposure (n = 806; 1.4%) playing lesser roles. The overall release rate for animals receiving care was 50.2% while 45.3% died or were euthanized during the rehabilitation process. A relatively small number (0.3%) were permanently non-releasable and placed in captivity; 4.1% had unknown outcomes. A comparable evaluation in 1989 revealed that wildlife submissions have increased (annual mean 12,583 vs 19,395), and are accompanied by a significant improvement in release (50.2% in the study period vs 44.4% in 1989) (χ^2^(1) = 90.43, p < 0.0001). In this manuscript, we aim to describe the rehabilitator community in New York State, and present the causes and outcomes for rehabilitation over a three-year period.

## Introduction

Ethical questions and skepticism over the ecological benefits have fueled debate on rehabilitative treatment of wild animals [1, 2]. Wildlife rehabilitation is the practice of caring for sick, injured, orphaned, and displaced wild animals with the primary goal of returning them to their natural habitat. The value of rehabilitation for individual animals is controversial, as some argue that stressors on animals undergoing care may be as traumatizing to the animal as the inciting event [3–5]. In contrast, advocates urge that the need for rehabilitation often arises from anthropogenic causes and humans, therefore, have a moral obligation to rectify their impact [6]. In addition, rehabilitation provides people with close contact with wildlife, potentially increasing knowledge of wild species and factors contributing to their declines, which can have positive impacts on local biodiversity conservation [6].

Despite debate, in the United States, wildlife rehabilitation is a publicly accepted practice in many regions and may be beneficial to state wildlife agencies as a public service. Wildlife rehabilitators, typically dedicated private citizens or small non-profit centers, may invest significant time and personal resources in this activity. Unlike rehabilitation in other parts of the world, this practice is typically minimally funded by governmental institutions and relies on personal donations of time and finances. The National Wildlife Rehabilitators Association (NWRA) reports that a rehabilitator volunteers an average of 32–36 hours per week through the spring and summer of each calendar year [7], which may indicate a large volume of animals being handled. The rehabilitator plays a primary role in the ultimate disposition of the animal in question, from selecting an appropriate release location to consulting on the euthanasia of individuals that cannot be rehabilitated. Additional multi-disciplinary roles include public outreach, education, advocacy, and collaboration with veterinary professionals.

Tangible benefits may be derived from data rehabilitators are responsible for collecting and reporting about the animals they care for. This information may be an underutilized resource that can contribute to the understanding of local and national populations, as well as the overall health and well-being of the habitat shared between wildlife and people [8]. Global declines in wildlife numbers raise important questions regarding specific causes of these trends. Recent studies estimate that one-third of terrestrial wildlife admissions to rehabilitation facilities and 70% of loggerhead sea turtle (*Caretta caretta*) stranding events that required rehabilitative intervention were attributed to anthropogenic activity [1, 9].

Part of the challenge with resolving conflicts, answering questions about impacts, or even improving basic treatment strategies is the limited data on wildlife rehabilitation to support informed decisions. In 1980, the New York State Department of Environmental Conservation (DEC) began issuing wildlife rehabilitation licenses to qualified private citizens. In New York State, wildlife rehabilitators achieve their Class I license following completion of a training course and written examination. These rehabilitators are licensed to treat non-migratory birds and most mammalian species. The ability to treat a wider range of species (including large mammals, migratory and protected bird species, rabies vector species, marine mammals) and graduation to a Class II license requires a minimum of two years as a Class I rehabilitator, as well as additional training and appropriate licensing [10]. Since 1985, rehabilitators of all levels have been required to contribute to the state’s knowledge of wildlife health by submitting annual reports on paper forms. Data that could provide information on rehabilitation caseloads, species affected, causes of presentation, and treatment outcomes/disposition were collected, but were not in a form conducive for such data summaries. We digitized archived paper records from a three-year time period (2012–2014). Previous analyses were available from 1989, so further comparison is possible to detect changes in the system over time.

Here, we report large-scale epidemiologic data to provide a foundation for understanding the scope and potential impacts of wildlife rehabilitation in New York State. The aims of this study are two-fold: (1) to describe the rehabilitator community in New York State, including the number of animals the citizens treat and how this has changed over time, and (2) to demonstrate the causes for which wild animals are presented for rehabilitation in New York State, as well as the likelihood of release based on this presenting distress and taxonomy. Previous publications examining wildlife rehabilitation have been limited to a single species or taxon, rehabilitation center, or cause of distress [11–15]. Here, while we highlight some specific examples of presentation and utilize species of interest to demonstrate the practice, we uniquely report *all* cases from the state of New York (~55,000mi^2^) in a three-year period, spanning multiple rehabilitators and hospitals and inclusive of all species and presenting complaints. This information is intended to assist rehabilitators in allocating limited treatment resources and improving care; and inform wildlife agencies tasked with oversight about the scope, needs, and impact of rehabilitation.

## Materials and methods

### Study design

A retrospective study of wildlife rehabilitation in the state of New York (USA) was performed using licensed wildlife rehabilitator logs submitted to the New York State Department of Environmental Conservation (NYSDEC). The state of New York is in the northeastern region of the United States and has a temperate climate. Most of the state’s land area is dominated by broadleaf and mixed forest types and agricultural land uses. In 2020, the state’s population was approximately 20.2 million [16] approximately two thirds of which was located within the New York metropolitan area along the Atlantic coast in the southeast.

### Data source and preparation

Licensed wildlife rehabilitators in New York are permitted to rehabilitate native and naturalized wildlife species originating in the state of New York. License conditions required wildlife rehabilitators to record case information on a weekly basis using the Wildlife Rehabilitator Log (WRL) paper form provided by the DEC. In addition to species and intake date, approximate age, sex (if known), and location in which the animal was found are recorded. Both the presenting distress and final disposition are noted for each animal. Annual summaries and all WRL forms are submitted to the agency by December 1 of each calendar year. Data on these handwritten logs from the three reporting years ending December 1, 2012, 2013, and 2014 were manually transcribed into digital spreadsheets, and records from all years were then combined into a single Microsoft Office Access® database (Microsoft Corporation, Redmond, Washington, USA, 90852). All database records were reviewed for accuracy and formatting consistency. Simple data entry errors by the wildlife rehabilitators (such as minor incorrect spelling of species or place names) were corrected. Species names were standardized using currently accepted taxonomic lists [17, 18]. Terms used for causes of presentation/distress and dispositions were taken from the DEC classifications on the forms. Incomplete and inaccurate records, as well as records for domestic and captive animals, were excluded from the analysis.

### Animal classification

Species reported were aggregated into six primary taxonomic groupings and 16 secondary groupings. These groups were created based on the following criteria: taxonomy, natural history, and commonalities in the way in which species are handled by the rehabilitation community. These groups are as follows: birds (columbiformes, raptors, passerines, waterfowl, and other), mammals (large mammals, small herbivores, and small carnivores), rabies vector species (bats, raccoons (*Procyon lotor*), and striped skunk (*Mephitis mephitis*)), reptiles (turtles, snakes, lizards), amphibians (frogs and salamanders) and unknown. The number of reptiles other than turtles were too few for meaningful analysis, so this category was reduced to turtles alone. A list of species included in the analysis for each grouping is provided.

### Cause of distress classification

On the WRL form, the causes of distress included 15 primary categories and 41 subcategories. For analytical purposes, the 15 primary categories were aggregated into six groupings: 1) orphaned (orphaned, orphaned due to unnecessary human intervention, developmental abnormality); 2) trauma (accidental entrapment, collision, entanglement, injured by other animal or human, mechanical injury due to gun/arrow/mower/trap); 3) infectious (bacterial infection, parasitism, viral disease); 4) poisoning/toxin (poison or toxin ingestion, soaked or similar damage); 5) habitat loss (human disturbance, i.e. tree-cutting, building construction; and natural disturbance, i.e. flood, fire, etc.); and 6) unknown. Because of the great interest in understanding the impact of domestic cats on declining wildlife numbers [19], trauma from cat attacks have been disaggregated and presented where appropriate. A small subset of records had more than one distress code provided. For these cases, each combination was evaluated, and one reported cause was selected as the primary reason for presentation to a rehabilitation facility based on the perceived order of events and relative detriment to an animal’s health condition. For example, all causes of trauma or poisoning/toxin were selected as primary when combined with orphaning, while parasitism was considered secondary to orphaning. This prioritization was determined and agreed upon by a committee made up of veterinarians, a wildlife biologist, and a data analyst.

### Disposition classification

The WRL disposition categories included: 1) died under/prior to care; 2) euthanized; 3) released; 4) still under care; 5) transferred to another licensed wildlife rehabilitator; 6) permanently non-releasable and transferred to a licensed person or educational institute; and 7) unknown. When cases were marked as being transferred to a second rehabilitator, only the secondary record was included in the analysis in order to avoid duplication. The disposition categories were combined into the following groups for analysis: 1) died (died under/prior to care, euthanized); 2) released; 3) non-releasable (placed under permanent care of licensed individual or educational institution); and 4) unknown (still under care or unknown).

### Analysis

Because licensed wildlife rehabilitators in the State of New York are required to submit annual case logs, the 2012–2014 dataset was effectively a complete census of all animals under the care of wildlife rehabilitators during that three-year time period. Our basic summary metrics for this census include proportions and counts and negate the need for inferential statistics.

Cases and release rates were reported for the most common species within each species grouping. Individual species that were anomalies or of interest in New York were identified in the results. For instance, bald eagles (*Haliaeetus leucocephalus*) were reported individually because of specific interest by the DEC on the scope of animals in rehabilitation relative to population estimates derived from the number of resident breeding pairs. Results were compared to a previous survey of wildlife rehabilitators performed in New York State in 1989 to investigate trends, progression, and changes that occurred over the past 25 years. The 1989 survey was published as an internal agency report for the DEC in order to evaluate wildlife rehabilitation activity in the state and the attitudes and perspectives of rehabilitators.

All statistical analyses were performed using GraphPad Prism version 7.05 for Windows (GraphPad Software, San Diego, California, USA).

## Results

Between 2012 and 2014, rehabilitators reported 58,185 wildlife rehabilitation cases to the DEC. The cases were primarily birds (n = 30,182; 51.9%) and mammals (n = 25,447; 43.7%) with lesser numbers of reptiles (n = 2,421; 4.2%) and amphibians (n = 75, 0.1%). The remainder (n = 60, 0.1%) were not clearly specified.

Over the course of the three-year study period, 696 uniquely identifiable rehabilitators submitted reports to the DEC. Each year, between 441 and 458 of these rehabilitators had at least one case and submitted their WRL. The majority of rehabilitators (74.7%) saw 25 or fewer animals per annum, accounting for 13.8% of cases. By comparison, 45.5% of all animals were seen by the 2.4% of rehabilitators who saw more than 300 animals annually. These results are compared to the demographics of wildlife rehabilitators in 1989 (Table 1). We found no evidence of a difference in the numbers of rehabilitators within caseload groupings between the 1989 reporting year and the 2014 reporting year (χ^2^(6) = 8.887, p = 0.18).

**Table 1:** Annual caseload reported by individual wildlife rehabilitators with at least one reported case in New York 1989 and 2012–2014.

	1989 ^a^	2012		2013		2014	
Annual Caseload	Rehabilitators	Rehabilitators	Cases	Rehabilitators	Cases	Rehabilitators	Cases
**1–25**	208 (68.0%)	331 (75.1%)	2,566	344 (74.9%)	2,782	340 (74.2%)	2,675
**26–50**	43 (14.1%)	46 (10.4%)	1,747	44 (9.6%)	1,582	51 (11.1%)	1,853
**51–100**	24 (7.8%)	34 (7.7%)	2,505	34 (7.4%)	2,445	30 (6.6%)	2,039
**101–150**	12 (3.9%)	11 (2.5%)	1,357	11 (2.4%)	1,331	12 (2.6%)	1,507
**151–200**	4 (1.3%)	5 (1.1%)	887	9 (2.0%)	1,467	11 (2.4%)	1,865
**201–300**	6 (2.0%)	4 (0.9%)	885	7 (1.5%)	1,722	2 (0.4%)	477
**301+**	8 (2.6%)	10 (2.3%)	6,076	10 (2.2%)	9,440	12 (2.6%)	10,977
**Total**	**306**	**441**	**16,023**	**459**	**20,769**	**458**	**21,393**

^a.^ Data included for comparison from Appendix E. Summary of animals handled, by DEC administrative region; by New York State wildlife rehabilitators in 1989. [13].

A cause of distress was provided for 85.4% of the records, with 2.3% of the cases having more than one cause of distress code given. When the cases were aggregated into the six general groupings, trauma was the most common overall cause of submission (38.1%) followed by orphaning (37.3%); habitat loss (6.8%); infectious disease (3.1%); and poisoning/toxin (1.4%). The remaining cases (13.4%) had an unknown cause of distress or did not provide a cause of distress.

Final dispositions were reported for 95.9% of cases. During the study period, 50.2% (29,227/58,185) of all animals presented were successfully released to the wild. This represents a significant improvement in release rate from 44.4% (5590/12583) in 1989 (χ^2^(1) = 90.43, p < 0.0001). Release rates varied significantly between taxonomic classes (χ^2^(3) = 330.4, p < 0.0001). Amphibians had the highest release rate (57.3%; 43/75) followed by mammals (54.5%; 13,868/25,447), reptiles (47.5%; 1,149/2,421), and birds (46.9%; 14,147/30182).

Rehabilitators reported that 26,385 animals (45.3%) either died or were euthanized during rehabilitation. Other dispositions were rare in comparison; 3.4% remained under the care of the rehabilitator at the time of reporting, and 0.3% were deemed permanently non-releasable. The non-releasable animals were then moved to an educational facility or placed under the care of a licensed professional at another location. No disposition information was provided for 441 cases (0.8%).

By species, waterfowl (Table 2) had the highest overall rate of release (65.9%) when compared with other groups. Of successfully released waterfowl, 53% had arrived at the facilities due to orphaning, while 22% had arrived as a result of trauma. The mallard duck (*Anas platyrhynchos*) had the highest overall release success rate (77.9%) of all species.

**Table 2:** Cause of presentation and corresponding disposition for waterfowl attended by New York state rehabilitators between 2012 and 2014.

	Habitat Loss		Infectious		Orphaned		Poisoning/Toxin		Trauma		Unknown		Total	
	Cases	% Rel.^a^	Cases	% Rel.	Cases	% Rel.	Cases	% Rel.	Cases	% Rel.	Cases	% Rel.	Cases	% Rel.
**Mallard** ^b^	32	66%	9	56%	1133	85%	11	91%	523	66%	310	73%	2,018	78%
**Canada Goose** ^c^ ^**.**^	9	56%	17	65%	244	87%	18	28%	411	42%	198	55%	897	57%
**Wood Duck** ^d^ ^.^	4	75%	0	0%	374	52%	0	0%	37	27%	28	57%	443	50%
**Diving Ducks** ^e^ ^.^	136	61%	1	0%	69	58%	3	0%	50	30%	76	55%	335	54%
**Other** ^f^ ^**.**^	17	65%	23	52%	32	81%	32	56%	137	39%	147	54%	388	52%
**Total**	**198**	**62%**	**50**	**56%**	**1,852**	**78%**	**64**	**52%**	**1,158**	**52%**	**759**	**62%**	**4,081**	**66%**

^a.^ % Rel = Percent Released.

^b.^ *Anas platyrhynchos*

^c.^ *Branta candensis*

^d.^*Aix sponsa*.

^e.^ Includes: black scoter (*Melanitta americana*), canvasback (*Aythya valisineria*), common merganser (*Mergus merganser*), greater scaup (*Aythya marila*), hooded merganser (*Lophodytes cucullatus*), lesser scaup (*Aythya affinis*), red-breasted merganser (*Mergus serrator*), redhead (*Aythya americana*), ring-necked duck (*Aythya collaris*), surf scoter (*Melanitta perspicillata*), and white-winged scoter (*Melanitta deglandi*).

^f.^ Includes: ducks: American black duck (*Anas rubripes*), American wigeon (*Mareca americana*), bufflehead (*Bucephala albeola*), common eider (*Somateria mollissima*), common goldeneye (*Bucephala clangula*), gadwall (*Mareca strepera*), long-tailed duck (*Clangula hyemalis*), northern shoveler (*Spatula clypeata*), ruddy duck (*Oxyura jamaicensis*), geese: brant (*Branta bernicla*), snow goose (*Anser caerulescens*); Swans: mute swan (*Cygnus olor*), trumpeter swan (*Cygnus buccinator*), and tundra swan (*Cygnus columbianus*).

Raptors (Table 3) had the lowest collective release rate, though release success varied greatly depending on reason for admittance; orphaning had an 84.3% release rate, while trauma had a 40.4% release rate. The influence of presenting distress on release success was consistent among raptor species, including the most commonly presenting species, the red-tailed hawk (*Buteo jamaicensis*). Trauma was the most common cause of distress for raptor species (57.0%), followed by unknown/unspecified causes (20.1%), and orphaning (12.3%). Shooting as a cause of admittance to rehabilitation was confirmed in 20 raptor cases, representing just over 1.0% of raptor trauma cases (20/1,831) with a 30% mortality rate (6/20).

**Table 3:** Cause of presentation and corresponding disposition for raptors attended by New York state rehabilitators between 2012 and 2014.

	Habitat Loss		Infectious		Orphaned		Poisoning/Toxin		Trauma		Unknown		Total	
	Cases	% Rel.^a^	Cases	% Rel.	Cases	% Rel.	Cases	% Rel.	Cases	% Rel.	Cases	% Rel.	Cases	% Rel.
**Red-tailed Hawk** ^b^ ^**.**^	23	70%	75	23%	34	76%	20	20%	539	37%	209	34%	900	37%
**E. Screech-Owl** ^c^ ^**.**^	34	79%	7	71%	113	84%	0	0%	267	47%	71	56%	492	60%
**American Kestrel** ^d^ ^**.**^	16	94%	1	100%	174	91%	2	50%	94	53%	41	51%	328	75%
**Barred Owl** ^e^ ^**.**^	8	38%	4	0%	16	81%	3	100%	257	51%	30	27%	318	50%
**Great Horned Owl** ^f^ ^**.**^	14	64%	19	26%	20	65%	7	29%	140	38%	73	37%	273	40%
**Cooper’s Hawk** ^g^ ^**.**^	7	71%	18	11%	8	100%	7	43%	180	37%	49	29%	269	37%
**Broad-winged Hawk** ^h^ ^**.**^	5	80%	5	20%	8	63%	1	0%	60	38%	13	38%	92	41%
**Sharp-shinned Hawk** ^i^ ^**.**^	1	100%	4	25%	0	0%	0	0%	56	38%	18	17%	79	33%
**Bald Eagle** ^j^ ^**.**^	5	80%	5	20%	3	67%	7	43%	14	36%	21	48%	55	45%
**Golden Eagle** ^k^ ^**.**^	0	0%	0	0%	0	0%	0	0%	0	0%	1	100%	1	100%
**Other** ^**l.**^	17	29%	14	7%	20	65%	11	18%	224	30%	119	36%	405	33%
**Total**	**130**	**68%**	**152**	**22%**	**396**	**84%**	**58**	**31%**	**1,831**	**40%**	**645**	**38%**	**3,212**	**45%**

^a^ % Rel = Percent Released.

^b.^ *Buteo jamaicensis*

^c.^ *Megascops asio*

^d^ *Falco sparverius*

^e.^ *Strix varia*

^f.^ *Bubo virginianus*

^g.^ *Accipiter cooperii*

^h.^ *Buteo platypterus*

^i.^ *Accipiter striatus*

^j.^ *Haliaeetus leucocephalus*

^k.^*Aquila chrysaetos*.

^i.^ Includes: barn owl (*Tyto alba*), black vulture (*Coragyps atratus*), golden eagle (*Aquila chrysaetos*), long-eared owl (*Asio otus*), merlin (*Falco columbarius*), northern goshawk (*Accipiter gentilis*), northern harrier (*Circus hudsonius*), northern saw-whet owl (*Aegolius acadicus*), osprey (*Pandion haliaetus*), peregrine falcon (*Falco peregrinus*), red-shouldered hawk (*Buteo lineatus*), rough-legged hawk (*Buteo lagopus*), short-eared owl (*Asio flammeus*), snowy owl (*Bubo scandiacus*), and turkey vulture (*Cathartes aura*).

Bald eagles represented less than 2% of raptors presented to wildlife rehabilitation centers. Approximately one-quarter of all admissions were a result of trauma, with a release rate of 36% closely mirroring that for other trauma-victim raptors. The next most commonly known cause for presentation of bald eagles was poisoning/toxin (12.7%). Bald eagles presented more commonly with this distress relative to any other raptor species included in this analysis, but only three of seven individuals were successfully rehabilitated. Only a single Golden Eagle was recorded during the study period, and was successfully released after presenting for an unknown cause.

Passerines (Table 4) were the second most likely group to be submitted for rehabilitation, behind small herbivores, and had an overall release rate of 46.1%. The American crow (*Corvus brachyrhynchos*) had the lowest overall release rate for any species evaluated in this study across almost all categories of distress. Crows presented to wildlife rehabilitation centers primarily for infectious disease 14.6% of the time, far more frequently than all other passerines (2.0%). These crows had lower release rates (3.8%) than all other passerines with infectious disease (31.0%).

**Table 4:** Cause of presentation and corresponding disposition for passerines attended by New York state rehabilitators between 2012 and 2014.

	Habitat Loss		Infectious		Orphaned		Poisoning/Toxin		Trauma		Unknown		Total	
	Cases	% Rel.^a^	Cases	% Rel.	Cases	% Rel.	Cases	% Rel.	Cases	% Rel.	Cases	% Rel.	Cases	% Rel.
American Robin^b^^.^	251	73%	46	26%	1,050	70%	19	26%	1,145	31%	493	41%	3,004	49%
Sparrow^c^^.^	205	53%	33	39%	944	50%	32	47%	762	30%	371	36%	2,347	42%
European Starling^d^^.^	272	81%	19	16%	761	69%	12	33%	369	32%	210	52%	1,643	60%
Blue Jay^e^^.^	23	74%	21	5%	195	63%	7	0%	309	30%	142	31%	697	40%
Common Grackle^f^^.^	34	44%	25	24%	200	49%	2	50%	187	24%	160	32%	608	36%
American Crow^g^^.^	11	27%	79	4%	78	63%	4	0%	225	22%	145	26%	542	26%
Other^h^^.^	308	66%	98	41%	1,006	57%	41	29%	1,891	40%	678	40%	4,022	46%
**Grand Total**	**1,104**	**68%**	**321**	**24%**	**4,234**	**61%**	**117**	**32%**	**4,888**	**34%**	**2,199**	**38%**	**12,863**	**46%**

^a.^ % Rel = Percent Released.

^b.^*Turdus migratorius*.

^c.^ Includes: American tree sparrow (*Spizelloides arborea*), chipping sparrow (*Spizella passerina*), field sparrow (*Spizella pusilla*), fox sparrow (*Passerella iliaca*), Harris’s sparrow (*Zonotrichia querula*), house sparrow (*Passer domesticus*), Lincoln’s sparrow (*Melospiza lincolnii*), Savannah sparrow (*Passerculus sandwichensis*), song sparrow (*Melospiza melodia*), swamp sparrow (*Melospiza georgiana*), vesper sparrow (*Pooecetes gramineus*), and white-throated sparrow (*Zonotrichia albicollis*).

^d.^Sturnus vulgaris

^e.^Cyanocitta cristata

^f.^Quiscalus quiscula

^g.^ Corvus brachyrhynchos.

^h.^ Includes: families Bombycillidae (waxwings), Calcariidae, Cardinalidae (cardinals), Certhiidae, Corvidae (corvids), Fringillidae (finches), Hirundinidae (swallows), Icteridae, Laniidae, Mimidae (mockingbirds and thrashers), Motacillidae, Paridae (chickadees and tits), Parulidae (warblers), Phylloscopidae, Polioptilidae, Regulidae (kinglets), Sittidae (nuthatches), Troglodytidae (wrens), Turdidae (thrushes), Tyrannidae (tyrant flycatchers), and Vireonidae (vireos).

The Columbiforme group (Table 5) was dominated by pigeons (*Columba* spp.), which were the most frequently treated birds in the study. Trauma was the most common cause of admittance for both Columbiformes and passerines, and pigeons had the highest release rates for trauma victims in both of these groups.

**Table 5:** Cause of presentation and corresponding disposition for pigeons and doves attended by New York state rehabilitators between 2012 and 2014.

	Habitat Loss		Infectious		Orphaned		Poisoning/Toxin		Trauma		Unknown		Total	
	Cases	% Rel.^a^	Cases	% Rel.	Cases	% Rel.	Cases	% Rel.	Cases	% Rel.	Cases	% Rel.	Cases	% Rel.
**Pigeon** ^b^	120	68%	900	38%	885	66%	407	48%	2,253	45%	1,005	39%	5,570	47%
**Dove** ^c^	35	80%	38	21%	259	68%	15	13%	804	32%	294	46%	1,445	42%
**Total**	**155**	**70%**	**938**	**37%**	**1,144**	**66%**	**422**	**46%**	**3,057**	**41%**	**1,299**	**40%**	**7,015**	**46%**

^a.^ % Rel = Percent Released.

^b.^ Includes: rock pigeon (*Columba livia*).

^c.^ Includes: mourning dove (*Zenaida macroura*) and ring-necked dove (*Streptopelia decaocto*).

The large mammal group (Table 6) were infrequently submitted to rehabilitation. Approximately half of all admissions were orphaned; 85.9% of the large mammals treated were neonates and juveniles, which was the highest proportion of all groups. Though trauma was not a common reason for admittance, large mammal trauma patients had a relatively low release rate. White-tailed deer were the most commonly presenting species in this group, yet had an overall release rate that was lower than all other large mammal species. Orphaned deer alone made up more than 1/3 of all large mammal admissions, but were less likely to be released than any other orphaned species in this group.

**Table 6:** Cause of presentation and corresponding disposition for large mammals attended by New York state rehabilitators between 2012 and 2014.

	Habitat Loss		Infectious		Orphaned		Poisoning/Toxin		Trauma		Unknown		Total	
	Cases	% Rel.^a^	Cases	% Rel.	Cases	% Rel.	Cases	% Rel.	Cases	% Rel.	Cases	% Rel.	Cases	% Rel.
**White-tailed Deer** ^b^ ^**.**^	9	44%	25	28%	578	55%	7	29%	443	26%	119	19%	1,181	40%
**American Black Bear** ^c^ ^**.**^	1	0%	0	0%	40	68%	0	0%	11	45%	1	100%	53	62%
**Eastern Coyote** ^d^ ^**.**^	0	0%	2	50%	27	59%	0	0%	6	50%	2	0%	37	54%
**Other** ^e^ ^**.**^	6	67%	54	43%	179	84%	6	0%	102	43%	53	49%	400	62%
**Total**	**16**	**50%**	**81**	**38%**	**824**	**62%**	**13**	**15%**	**562**	**29%**	**175**	**29%**	**1,671**	**46%**

^a.^ % Rel = Percent Released.

^b.^ *Odocoileus virginianus*

^c.^ *Ursus americanus*

^d.^*Canis latrans x Canis lycaon*.

^e.^ Includes: bobcat (*Lynx rufus*), gray fox (*Urocyon cinereoargenteus*), and red fox (*Vulpes vulpes*).

The small herbivore group (Table 7) contained the species most commonly submitted for rehabilitation, with the majority (87.4%) being eastern cottontails (*Sylvilagus floridanus*) and eastern grey squirrels (*Scurius carolinensis*). The overall release rates for grey squirrels and eastern cottontails were 65.4% and 44.7%, respectively. The leading primary cause of distress for eastern cottontails was trauma (50.5%), which was typically associated with a poor outcome, indicated by a release rate of just 33.7%. More specifically, 2,807 rabbits (31.8% of all admissions for the species) were associated with a domestic dog or cat attacks. Trauma (18.3%) was much less common in grey squirrels, whereas orphaning (59.8%) was the leading cause of distress. The release rate for orphaned squirrels was higher (75.0%) than that for eastern cottontails (58.3%).

**Table 7:** Cause of presentation and corresponding disposition for small herbivores attended by New York state rehabilitators between 2012 and 2014.

	Habitat Loss		Infectious		Orphaned		Poisoning/Toxin		Trauma		Unknown		Total	
	Cases	% Rel.^a^	Cases	% Rel.	Cases	% Rel.	Cases	% Rel.	Cases	% Rel.	Cases	% Rel.	Cases	% Rel.
**E. Cottontail** ^b^ ^**.**^	472	53%	27	15%	3,452	58%	36	31%	4,451	34%	384	43%	8,822	45%
**E. Gray Squirrel** ^c^ ^**.**^	981	73%	93	28%	4,441	75%	36	44%	1,358	39%	519	44%	7,428	65%
**Other Squirrel Sp.** ^d^ ^**.**^	129	61%	3	33%	363	66%	2	0%	164	60%	47	60%	708	63%
**Woodchuck** ^e^ ^**.**^	3	67%	9	33%	191	82%	4	0%	188	46%	70	40%	465	60%
**Other** ^f^ ^**.**^	120	62%	12	25%	433	44%	24	17%	453	37%	128	29%	1,170	41%
**Total**	**1,705**	**66%**	**144**	**26%**	**8,880**	**67%**	**102**	**30%**	**6,614**	**36%**	**1,148**	**42%**	**18,593**	**54%**

^a.^ % Rel = Percent Released.

^b.^ *Sylvilagus floridanus*

^c.^*Sciurus carolinensis*.

^d.^ Includes: fox squirrel (*Sciurus niger*), northern flying squirrel (*Glaucomys sabrinus*), red squirrel (*Sciurus vulgaris*), and southern flying squirrel (*Glaucomys volans*).

^e.^*Marmota monax*.

^f.^ Includes: orders Lagomorpha, Rodentia, and Eulipotyphla. Additional Lagomorpha species include: snowshoe hare (*Lepus americanus*). Additional Eulipotyphla species include: eastern mole (*Scalopus aquaticus*), northern short-tailed shrew (*Blarina brevicauda*), and star-nosed mole (*Condylura cristata*). Additional Rodentia species include: American beaver (*Castor canadensis*), black rat (*Rattus rattus*), brown rat (*Rattus norvegicus*), common vole (*Microtus arvalis*), deer mouse (*Peromyscus*) sp., eastern chipmunk (*Tamias striatus*), eastern woodrat (*Neotoma floridana*), house mouse (*Mus musculus*), meadow jumping mouse (*Zapus hudsonius*), meadow vole (*Microtus pennsylvanicus*), muskrat (*Ondatra zibethicus*), North American porcupine (*Erethizon dorsatum*), southern red-backed vole (*Myodes gapperi*), white-footed mouse (*Peromyscus leucopus*), woodland jumping mouse (*Napaeozapus insignis*), and woodland vole (*Microtus pinetorum*).

Rabies vector species (Table 8) were most commonly submitted to wildlife rehabilitators for orphaning, but were otherwise infrequently treated compared to other mammals. Although raccoons are common across New York State, only 7.3% of all mammals treated were raccoons; skunks and bats together accounted for less than 3% of all mammals treated. Particularly low numbers of admissions were recorded for the little brown bat (*Myotis lucifugus*) (n = 38), which was once the most populous bat species in the state.

**Table 8:** Cause of presentation and corresponding disposition for rabies vector species attended by New York state rehabilitators between 2012 and 2014.

	Habitat Loss		Infectious		Orphaned		Poisoning/Toxin		Trauma		Unknown		Total	
	Cases	% Rel.^a^	Cases	% Rel.	Cases	% Rel.	Cases	% Rel.	Cases	% Rel.	Cases	% Rel.	Cases	% Rel.
**Raccoon** ^b^ ^.^	71	48%	33	0%	1384	64%	8	13%	187	43%	169	57%	1,852	59%
**Striped Skunk** ^c^ ^.^	0	0%	15	7%	286	89%	1	0%	54	17%	46	50%	402	71%
**Bats** ^d^ ^.^	14	57%	10	0%	50	50%	2	50%	152	60%	107	36%	335	49%
**Total**	**85**	**49%**	**58**	**2%**	**1,720**	**67%**	**11**	**18%**	**393**	**46%**	**322**	**49%**	**2,589**	**60%**

^a.^ % Rel = Percent Released.

^b.^ *Procyon lotor*

^c.^*Mephitis mephitis*.

^d.^ Includes: silver-haired bat (*Lasionycteris noctivagans*), eastern red bat (*Lasiurus borealis*), little brown bat (*Myotis lucifugus*), big brown bat (*Eptesicus fuscus*), hoary bat (*Lasiurus cinereus*), northern long-eared bat (*Nyctophilus arnhemensis*).

The small carnivore group (Table 9) was comprised almost entirely of the Virginia opossum (*Didelphis virginiana*). This species most frequently presented as orphaned with a 63.8% release rate, and an overall release rate of 60.6%.

**Table 9:** Cause of presentation and corresponding disposition for small carnivores attended by New York state rehabilitators between 2012 and 2014.

	Habitat Loss		Infectious		Orphaned		Poisoning/Toxin		Trauma		Unknown		Total	
	Cases	% Rel.^a^	Cases	% Rel.	Cases	% Rel.	Cases	% Rel.	Cases	% Rel.	Cases	% Rel.	Cases	% Rel.
**Virginia Opossum** ^b^ ^**.**^	8	75%	18	17%	1795	64%	11	9%	531	54%	157	54%	2,520	61%
**Other** ^c^ ^**.**^	0	0%	3	33%	22	55%	0	0%	36	50%	8	25%	69	48%
**Total**	**8**	**75%**	**21**	**19%**	**1,817**	**64%**	**11**	**9%**	**567**	**53%**	**165**	**53%**	**2,589**	**60%**

^a.^ % Rel = Percent Released.

^b.^*Didelphis virginiana*.

^c.^ Includes: American marten (*Martes americana*), American mink (*Neovison vison*), fisher (*Pekania pennanti*), least weasel (*Mustela nivalis*), long-tailed weasel (*Mustela frenata*), North American otter (*Lontra canadensis*), short-tailed weasel (*Mustela erminea*), and weasel (Mustela sp.).

Non-chelonian herpetofauna were infrequently reported. Turtles presented most often for trauma (53.1%), for which they had a release rate of 40.9%. Trauma due to collision with a car alone was responsible for 746 admissions, accounting for 32.7% of all admitted turtles. Rehabilitators also reported turtles as “orphans” 22.4% of the time, which had the lowest release rate for this distress category amongst the nine groups (Table 10).

**Table 10:** Cause of presentation and corresponding disposition for turtles attended by New York state rehabilitators between 2012 and 2014.

	Habitat Loss		Infectious		Orphaned		Poisoning/Toxin		Trauma		Unknown		Total	
	Cases	% Rel.^a^	Cases	% Rel.	Cases	% Rel.	Cases	% Rel.	Cases	% Rel.	Cases	% Rel.	Cases	% Rel.
**Comm. Box Turtle** ^b^ ^**.**^	128	56%	66	59%	273	31%	1	0%	464	29%	126	40%	1,058	36%
**Snapping turtle** ^c^ ^**.**^	60	88%	13	54%	90	70%	3	0%	359	58%	51	73%	576	64%
**Painted Turtle** ^d^ ^**.**^	22	82%	4	25%	92	73%	1	100%	314	39%	26	54%	459	48%
**Other** ^e^ ^**.**^	17	29%	10	30%	56	55%	0	0%	73	40%	29	24%	185	41%
**Total**	**227**	**65%**	**93**	**54%**	**511**	**48%**	**5**	**20%**	**1,210**	**41%**	**232**	**47%**	**2,278**	**46%**

^a.^ % Rel = Percent Released.

^b.^ *Terrapene Carolina*

^c.^ *Chelydra serpentina*

^d.^*Chrysemys picta*.

^e.^ Includes: Blanding’s turtle (*Emys blandingii*) or (*Emydoidea blandingii*), bog turtle (*Glyptemys muhlenbergii*), common musk turtle (*Sternotherus odoratus*), diamondback terrapin (*Malaclemys terrapin*), eastern mud turtle (*Kinosternon subrubrum*), spiny softshell turtle (*Apalone spinifera*), spotted turtle (*Clemmys guttata*), and wood turtle (*Glyptemys insculpta*).

Box turtles comprised 43.7% of all reptilian submissions, which presented most often as a result of trauma. These turtles were successfully released 29% of the time. Young, “orphaned” box turtles were the next largest group, with a similar release rate of 31%. Vehicular-related injury accounted for 19.4% of all box turtle trauma with a release rate of 28.1%.

Known domestic cat attacks were responsible for 3,936 cases of wildlife rehabilitation during the study period. An additional 4,500 animals were presented to rehabilitation centers with predator-induced wounds without a known perpetrator. Domestic cat attacks accounted for nearly twice the number of attacks by domestic dogs (2,060), and nearly 5 times the number of attacks by natural predators (824). Mammals (2,001), mostly belonging to the small herbivore group (1,954), were the most frequent victims of domestic cat attacks. Nearly 1,900 birds fell victim to the domestic cat, with 1,372 belonging to the passerine group. Wildlife known to have been injured by cats were successfully released 31% of the time, a loss of over 2,700 animals in a three-year span.

## Discussion

Wildlife rehabilitators handle thousands of animals annually. Understanding basic information by species, cause of distress, and likelihood of release is valuable not only to the rehabilitation community, but to state wildlife agencies responsible for managing this engaged stakeholder group [20]. Previous wildlife rehabilitation literature has typically been limited to single species, diagnosis, or wildlife rehabilitation center of interest [11–15]. A strength of the current study and the DEC dataset is that the data encompass all cases across the entire state over a multiple year period by licensed rehabilitators. This information can provide a foundation for regulatory management, including where the state can focus rehabilitator educational efforts, determining allocation of resources benefiting specific species, and improving public outreach on anthropogenic impacts, such as free-roaming cats.

While traditional interactions between humans and wildlife in the form of hunting and trapping have declined, the rise in wildlife rehabilitation demonstrates alternative ways the public engages with wildlife [20–22]. Wildlife rehabilitators are an avenue for the public to obtain information about native wildlife. Increasing awareness of defaunation and mass extinction [23, 24] may motivate more individuals to “save” wildlife on a local level. In New York, the annual case numbers have increased substantially since 1989. Increasing case numbers might reflect citizens’ increased awareness of wildlife species loss and willingness to engage with injured/orphaned wildlife. Alternatively, increasing case numbers might reflect higher human-wildlife interactions due to habitat fragmentation or increasingly distressed wildlife.

Agencies are tasked with managing the impacts and conflicts from wildlife rehabilitation, but may not have specific resources to address issues [3, 8, 25]. The number of wildlife rehabilitators increased between 1989 and 2012–2014. However, most rehabilitators handled 25 or less cases per year. A smaller fraction, less than 3%, handled more than 300 cases annually [26]. Interestingly, this demographic has not significantly changed since 1989, despite the growing caseload and improvement in release rate. It appears that within the rehabilitation community, a majority of the animals are cared for by a small number of centers; therefore, significant data collection, communication, and outreach can be efficiently accomplished by targeting those centers.

The majority of animals presented to rehabilitators were impacted by trauma and orphaning, much of which was likely anthropogenic. Approximately 1,000 animals each year were presented to NY rehabilitators as orphans due to confirmed unnecessary human intervention. These “orphaned” animals are frequently healthy and are mistakenly taken for rehabilitation by well-meaning members of the public when they are in fact being cared for by a parent [27, 28]. Improving public outreach regarding causes of wildlife injury and natural behaviors may help to reduce these numbers.

Evaluation of release rates by cause of distress and species provides useful information to rehabilitators or veterinarians to determine the likelihood of successful rehabilitation for an individual case or allocate resources. Of all animals admitted due to orphaning, 65.6% were successfully released to the wild. In contrast, only 37.3% of trauma cases were successfully released to the wild with considerable species differences in release rates. For example, waterfowl as a group had the highest collective release rate, including that for trauma victims.

For raptors, causes of distress documented by rehabilitators were consistent with previously published literature, which cited trauma as the most frequent cause of admission and mortality [29, 30]. However, unlike previously published literature, gunshot was infrequently reported for this group (1.1% of trauma causes) and associated with only a 30% mortality (6/20). Electrocution has also been previously demonstrated as a major cause of raptor mortality, but was unable to be evaluated retrospectively in our study as the DEC does not list this as a possible cause of distress on the rehabilitator log form [31]. This discrepancy from previous reports may be secondary to the need for further diagnostics, such as radiographs, to confirm the presence of shot in an animal, and as such it is reasonable to assume that some of these cases may have been absorbed into the general trauma category. Raptors typically require high standards of function to be deemed releasable, with two perfectly functioning wings, intact talons, and unobscured bilateral vision for diurnal species, which may contribute to their lower release rates for traumatic injuries. Data regarding release success for specific injuries (i.e. humeral fractures, ocular damage, shoulder luxation, etc.) are lacking and often lead to debate regarding the suitability of an animal for release. Collection of more detailed data regarding these injuries, treatments, and time spent in rehabilitation from these cases could be beneficial in further improving decision making.

For locally threatened or endangered species, rehabilitation of individuals may be of importance when the wild population is limited. Bald eagle populations have been closely monitored in New York since species restoration efforts began in 1976. In 2015, 264 breeding pairs were estimated statewide; consequently, the 55 eagles seen in rehabilitation during this study period may represent a significant portion of the population [32]. The iconic nature of bald eagles makes them a prime candidate for citizens to submit to rehabilitation facilities if they are found injured or distressed. Rehabilitation of bald eagles is particularly challenging due to the species’ propensity for developing secondary complications. Bald eagles are highly susceptible to stress-related injuries, as well as aspergillosis-associated respiratory disease and staphylococcal pododermatitis [33–35]. Yet, the bald eagle had an overall release rate that was similar to other raptors, (45.5%), perhaps due to the significant resources dedicated to their intake and care. Bald eagles presented more frequently for poisoning/toxin than any other raptor species. This may represent the impact of lead toxicity, which has historically affected the species heavily due to its scavenging feeding habits and position at the top of the food web [31, 36]. Recent post-mortem studies estimate approximately 17% of bald eagles from New York State to have resulted from lead toxicity, and over 80% of all deceased eagles to have had some exposure [37]. Over one-third of the bald eagles in our study population were presented to rehabilitation centers for an unknown cause. It is possible some of these admissions may have resulted from lead toxicity because the clinical signs can be vague, varying from malaise to profound neurologic consequences [38]. While lead analyzers are more frequently available in wildlife hospitals, they are expensive and such specialized equipment is not be readily available to most rehabilitators and veterinarians [39]. Toxicity-related presentations demonstrate potential large-scale health issues that may impacts species at the population level.

In New York, box turtles are listed as a species of special concern [40, 41]. Population estimates are not available for the species in New York State, likely due to the difficulty of surveying elusive and solitary reptiles, however successful surveys have been performed in neighboring states [42]. The frequency of vehicular trauma in box turtles presented to wildlife rehabilitators suggests that roadway construction and an increasing density of vehicular traffic are directly impacting this species. While box turtles were the most commonly presented reptile following vehicular trauma, the common snapping turtle (*Chelydra serpentina*) and painted turtle (*Chrysemys picta*) were also frequent victims. Interestingly, aquatic turtles were successfully rehabilitated following vehicular trauma more frequently than the box turtle, for reasons that are unclear. The species’ poor release rate emphasizes the importance of motorist education and habitat protection for this state-recognized species.

Wildlife rehabilitation data may help to address controversial topics in ecologic health and public policy. The impact of free-roaming domestic cats on wildlife populations has been heavily debated [1, 11, 12, 27, 28, 43]. We demonstrated nearly 4,000 animals known to be injured by cats over three years, with an additional 4,500 animals with wounds possibly inflicted by a predator. Known domestic cat attacks accounted for 6.8% of all wildlife rehabilitation cases in New York State. Previous studies have reported up to 14% of all admissions to rehabilitation centers to be a result of domestic pet interaction [28]. Less than one-third of cat-attack victims were successfully released, which closely mirrored prior literature describing the release rates for birds attacked by cats to be as low as 12.9% [27]. Additionally, species less commonly considered to be victims of cats, including lizards and bats, have been documented to suffer substantially from feline predation [11, 12]. In addition to birds and small mammals, our study documented several species of bats, turtles, and snakes to be confirmed victims of domestic cat predation. Quantitative evidence for the debilitating impact that free-ranging domestic cats have on wildlife may be impactful in public education and policy regulation.

Since wildlife rehabilitators handle large numbers of animals, we are able to track trends in species over time as an indicator of disease activity or population health. The American crow, known to suffer high mortality from West Nile Virus (WNV) [44–47], had the lowest overall release rate of any species. Real-time case reporting since the introduction of WNV in 1999 would have detected increased crow mortality, while annual case tracking combined with confirmatory testing could help elucidate ecological factors influencing WNV prevalence. Similarly, the small number of bats rehabilitated likely reflects the emerging fungal disease known as White Nose Syndrome (WNS). Previously New York’s most common bat species, the little brown bat population has been devastated by WNS since its arrival in 2006; the population has experienced a 90% decrease and has the potential for extinction by 2026 [48, 49]. Submissions to the state’s rabies laboratory also show a precipitous decline in this species over the same time period [50].

Trained rehabilitators serve as an important buffer between the public and wildlife disease threats. When rehabilitation is restricted or prohibited, members of the public may be reticent to abandon wild animals or submit them for immediate euthanasia. These animals may then be raised in inappropriate conditions, increasing to possibility of zoonotic disease transmission. Public health agencies can be tasked with animal confiscation while conducting expensive and time-consuming follow-up to control zoonotic disease exposures, such as rabies. In New York, additional requirements are necessary to obtain a license to rehabilitate rabies vector species. These include a minimum of two years’ experience as a rehabilitator, pre-exposure rabies vaccinations, attendance at an annual training session, additional record-keeping responsibilities, and an inspection of the rehabilitation facility by the state’s Department of Agriculture and Markets [10]. Over 2,500 raccoons, skunks, and bats were submitted for rehabilitation during the reporting period so the potential exposure to the public may be substantial if trained rehabilitators were not available.

We recognize several limitations to the current study. While the records represent all wildlife rehabilitation submissions over a three-year span, reporting errors and inconsistencies both within and between rehabilitation centers occurred. These ranged from spelling errors, to misidentified species, to failure to accurately report distress causes and final disposition. While selecting the primary distress for each case was done systematically for every possible combination, the process represents potential for bias from the authors. Finally, we define rehabilitation success as release to the wild. While true success may be defined as normal function and survival after the time of release, this information is unfortunately not available for most species on a large-scale post-rehabilitation. The lack of case follow-up is an inherent shortcoming in the current process of wildlife rehabilitation.

Wildlife rehabilitation plays an important role in public education and outreach about native wildlife. There are benefits for animal welfare, disease monitoring, and conservation [51–54]. Analysis of large-scale rehabilitation data can improve resource allocation, treatment methods, surveillance, public education, and regulatory decision making. Standardizing rehabilitator reporting and digital data collection would facilitate compilation and analysis to benefit both rehabilitators and the state agency regulators.

## Supporting information

S1 File Wildlife rehabilitation log. This form is provided by the NYSDEC to wildlife rehabilitators to assist in record-keeping.
(PDF)

S2 File Wildlife rehabilitation log tally. This form is provided by the NYSDEC to wildlife rehabilitators and includes all of the possible distress and disposition codes that can be reported. Its submission is mandatory by the December 1 of the reporting year.
(PDF)
