# Supplementary material for: Species, causes, and outcomes of wildlife rehabilitation in New York State
Source: PLoS One. 2021 Sep 21;16(9):e0257675. doi: 10.1371/journal.pone.0257675 (PMC8454955; doi:10.1371/journal.pone.0257675)
Supplement: S1 File — This form is provided by the NYSDEC to wildlife rehabilitators to assist in record-keeping. (PDF) [file pone.0257675.s001.pdf]

NEW YORK STATE OF OPPORTUNITY | Department of Environmental Conservation

Wildlife Rehabilitation Log

For more information on this license visit www.dec.ny.gov/permits/25027.html

*PART A (Please type or print all information)

name / date of birth

Last ______ First ______ M.I. ______ DOB (mm/dd/yyyy) ______

address

Street Address ______ Apartment/Unit ______ City ______

County ______ State ______ Zip Code ______

email / telephone

Email ______ Telephone (______) ______ - ______

NYS license # / fed permit # / fed permit expire

NYS License Number ______ Federal Permit Number ______ Federal Permit Expiration Date ______

Do you want your name to appear on the statewide list of Wildlife Rehabilitators? ☐ Yes ☐ No

*PART B (Please type or print all information)

SPECIES ______ # ADULTS ______ # JUVENILE ______ # UNKNOWN ______ TOTAL ______

FROM ______ DATE RECEIVED ______

LOCATION FOUND ______ CAUSE OF DISTRESS ______ ☐ Yes ☐ No VET CONSULTED

DISPOSITION	#R	#P	#T	#PC	#I	#D	#E	TOTAL

DATE ______ TRANSFERRED TO ______ NYS LICENSE NUMBER ______

SPECIES ______ # ADULTS ______ # JUVENILE ______ # UNKNOWN ______ TOTAL ______

FROM ______ DATE RECEIVED ______

LOCATION FOUND ______ CAUSE OF DISTRESS ______ ☐ Yes ☐ No VET CONSULTED

DISPOSITION	#R	#P	#T	#PC	#I	#D	#E	TOTAL

DATE ______ TRANSFERRED TO ______ NYS LICENSE NUMBER ______

SPECIES ______ # ADULTS ______ # JUVENILE ______ # UNKNOWN ______ TOTAL ______

FROM ______ DATE RECEIVED ______

LOCATION FOUND ______ CAUSE OF DISTRESS ______ ☐ Yes ☐ No VET CONSULTED

DISPOSITION	#R	#P	#T	#PC	#I	#D	#E	TOTAL

DATE ______ TRANSFERRED TO ______ NYS LICENSE NUMBER ______

NEW YORK STATE OF OPPORTUNITY | Department of Environmental Conservation

APPLICANT

Last ______ First ______ M.I. ______

*PART B Cont'd (Please type or print all information)

SPECIES ______ # ADULTS ______ # JUVENILE ______ # UNKNOWN ______ TOTAL ______

FROM ______ DATE RECEIVED ______

LOCATION FOUND ______ CAUSE OF DISTRESS ______ VET CONSULTED ☐ Yes ☐ No

DISPOSITION	#R	#P	#T	#PC	#I	#D	#E	TOTAL

DATE ______ TRANSFERRED TO ______ NYS LICENSE NUMBER ______

SPECIES ______ # ADULTS ______ # JUVENILE ______ # UNKNOWN ______ TOTAL ______

FROM ______ DATE RECEIVED ______

LOCATION FOUND ______ CAUSE OF DISTRESS ______ VET CONSULTED ☐ Yes ☐ No

DISPOSITION	#R	#P	#T	#PC	#I	#D	#E	TOTAL

DATE ______ TRANSFERRED TO ______ NYS LICENSE NUMBER ______

SPECIES ______ # ADULTS ______ # JUVENILE ______ # UNKNOWN ______ TOTAL ______

FROM ______ DATE RECEIVED ______

LOCATION FOUND ______ CAUSE OF DISTRESS ______ VET CONSULTED ☐ Yes ☐ No

DISPOSITION	#R	#P	#T	#PC	#I	#D	#E	TOTAL

DATE ______ TRANSFERRED TO ______ NYS LICENSE NUMBER ______

SPECIES ______ # ADULTS ______ # JUVENILE ______ # UNKNOWN ______ TOTAL ______

FROM ______ DATE RECEIVED ______

LOCATION FOUND ______ CAUSE OF DISTRESS ______ VET CONSULTED ☐ Yes ☐ No

DISPOSITION	#R	#P	#T	#PC	#I	#D	#E	TOTAL

DATE ______ TRANSFERRED TO ______ NYS LICENSE NUMBER ______

COMMENTS
