# Supplementary material for: Species, causes, and outcomes of wildlife rehabilitation in New York State
Source: PLoS One. 2021 Sep 21;16(9):e0257675. doi: 10.1371/journal.pone.0257675 (PMC8454955; doi:10.1371/journal.pone.0257675)
Supplement: S2 File — This form is provided by the NYSDEC to wildlife rehabilitators and includes all of the possible distress and disposition codes that can be reported. Its submission is mandatory by the December 1 of the reporting year. (PDF) [file pone.0257675.s002.pdf]

NEW YORK STATE OF OPPORTUNITY | Department of Environmental Conservation

Wildlife Rehabilitator Log Tally

For more information on this license visit www.dec.ny.gov/permits/25027.html

1. *LICENSEE INFORMATION

name / date of birth

______________________ Last   ______________________ First   ________ M.I.   ____________ DOB (mm/dd/yyyy)

address

______________________ Street Address   ________ Apartment/Unit   ______________________ City

______________________ County   ________ State   ____________ Zip Code

email / telephone

______________________ Email   (________) ________ - ________ Telephone

license / permit #'s / permit expiration date

______________________ NYS License #   ______________________ Federal Permit #   ______________________ Federal Permit Expiration Date

Do you want your name to appear on the statewide list of Wildlife Rehabilitators? ○ **Yes** ○ **No**

2. *HOUSING AND ANIMAL SPECIALIZATION

species accepted (check all that apply)
note: to rehabilitate raptors, waterfowl, and most passerines you must have a federal permit

☐ Large Mammals ☐ Small Mammals ☐ Raptors ☐ Reptiles & Amphibians ☐ Passerines ☐ Game Birds ☐ Waterfowl

specialized caging (check all that apply)

☐ Neonatal Care ☐ Juveniles ☐ Adults ☐ Emergency Care ☐ Pre Release ☐ Soft Release

3. DISPOSITION OF WILDLIFE

Summarize the information from your Wildlife Rehabilitation Logs. Provide the totals in the appropriate box and columns.

CODE AND DISPOSITION		BIRDS	MAMMALS	REPTILES	AMPHIBIANS
R	Released to the Wildlife				
P	Disposition pending, still under my care				
T	Transferred to another Wildlife Rehabilitator for continued care				
PC	Permanently non-releasable, transferred to NYS licensed person				
I	Permanently non-releasable, transferred to NYS licensed Ed Institute				
D	Died under care or prior to receiving care				
E	Euthanized				
	**TOTALS**				

Name

4. DISTRESS LETTER CODE

Code	Description	BIRDS	MAMMALS	REPTILES	AMPHIBIANS
A-1	PARENTS WHEREABOUTS UNKNOWN				
A-2	PARENTS KILLED BY DOMESTIC PET				
A-3	PARENTS KNOWN KILLED BY CAR				
A-4	UNNECESSARY HUMAN INTERVENTION				
B-1	COLLISION W/VEHICLE				
B-2	COLLISION W/WINDOW OR BUILDING				
B-3	COLLISION W/PHONE OR POWER LINE				
C-1	INJURED BY CAR				
C-2	INJURED BY DOG				
C-3	INJURED BY HUMAN				
C-4	INJURED BY NATURAL PREDATOR				
C-5	UNKNOWN				
D-1	SHOT (GUN OR ARROW)				
D-2	TRAP INJURY				
D-3	MOWER / TILLER / HAY BALER INJURY				
D-4	OTHER (PROVIDE TOTAL NUMBER)				
E-1	ENTRAPPED IN BUILDING				
E-2	ENTRAPPED IN FIREPLACE / CHIMNEY				
E-3	ENTRAPPED IN WINDOW-WELL				
E-4	HEAD IN JAR / CAN				
F-1	TANGLED IN FISHING LINE				
F-2	TANGLED IN BEVERAGE HOLDER				
F-3	TANGLED IN STRING OR WIRE OR OTHER				

DISTRESS LETTER CODE Cont'd

Code	Description	BIRDS	MAMMALS	REPTILES	AMPHIBIANS
G-1	OIL				
G-2	GAS				
G-3	OTHER (PROVIDE TOTAL NUMBER				
G-4	UNKNOWN				
H-1	INGESTION OF LEAD (SUSPECTED)				
H-2	INGESTION OF LAWN CHEMICALS				
H-3	INGESTION OF ANTIFREEZE				
H-4	OTHER (PROVIDE TOTAL NUMBER)				
H-5	UNKNOWN				
I-1	INTERNAL PARASITES				
I-2	EXTERNAL PARASITES				
J	DEVELOPMENTAL ANOMALY				
K	BACTERIAL INFECTION (SUSPECTED)				
L-1	DISTEMPER SUSPECTED)				
L-2	AVIAN POX (SUSPECTED)				
L-3	OTHER (PROVIDE TOTAL NUMBER)				
L-4	UNKNOWN				
M	NATURAL DISTURBANCE				
N-1	NEST REMOVED / TREE CUT				
N-2	BUILDING OR CONSTRUCTION				
N-3	OTHER (PROVIDE TOTAL NUMBER)				
O	OTHER (PROVIDE TOTAL NUMBER)				
P	UNKNOWN (PROVIDE TOTAL NUMBER)				

You must provide the original copies of your "annual wildlife rehabilitation log tally" sheet together with your wildlife rehabilitation logs at the end of the license year to initiate the renewal of your license. These documents are due on or before December 1, of the license year.

***NOTICE:** Pursuant to ECL Section 3-0301(2)(Q), false statements made on this application are punishable in accordance to Section 210.45 of the New York State Penal Law.

Licensee's Signature ______________________

Date ______________________
